# Evaluating different models of maternal stress on stress-responsive systems in prepubertal mice

**DOI:** 10.3389/fnins.2023.1292642

**Published:** 2023-12-07

**Authors:** Julietta A. Sheng, Robert J. Handa, Stuart A. Tobet

**Affiliations:** ^1^Biomedical Sciences, Colorado State University, Fort Collins, CO, United States; ^2^Department of Psychiatry, Mass General Hospital, Harvard Medical School, Boston, MA, United States; ^3^School of Biomedical Engineering, Colorado State University, Fort Collins, CO, United States; ^4^Innovation Center on Sex Differences in Medicine, Mass General Hospital, Cambridge, MA, United States

**Keywords:** stress, pregnancy, depression, behavior, maternal, social behavior, prepuberty

## Abstract

**Introduction:**

Maternal adversity during pregnancy influences neurodevelopment in human and model animal offspring. Adversity can result from stressors coming from many different directions ranging from environmental to nutritional and physiological to immune (e.g., infection). Most stressors result in fetal overexposure to glucocorticoids that have been directly linked to long- and short-term negative impacts on neurological health of offspring. Neuropsychiatric diseases postulated to have fetal origins are diverse and include such things cardiovascular disease, obesity, affective disorders, and metabolic and immune disorders.

**Methods:**

The experiments in the current study compare 3 stressors: prenatal exposure to dexamethasone (DEX), maternal high fat diet (HFD), and maternal caloric restriction (CR). Offspring of mothers with these treatments were examined prepubertally to evaluate stress responsiveness and stress-related behaviors in in male and female mice.

**Results:**

Prenatal exposure to synthetic glucocorticoid, DEX, resulted in decreased neonatal body weights, reduced social interaction behavior, and hypoactive stress response offspring exposed to maternal DEX. Maternal CR resulted in decreased body weights and social interaction behavior in males and females and increased anxiety-like behavior and acute stress response only in males. HFD resulted in altered body weight gain in both sex offspring with decreased anxiety-like behavior in a female-biased manner.

**Discussion:**

The idea that glucocorticoid responses to different stressors might serve as a common stimulus across stress paradigms is insufficient, given that different modes of prenatal stress produced differential effects. Opposite nutritional stressors produced similar outcomes for anxiety-like behavior in both sexes, social-like behavior in females, and a hyperactive adrenal stress response in males. One common theme among the three models of maternal stress (DEX, CR, and HFD) was consistent data showing their role in activating the maternal and fetal immune response. By tuning in on the more immediate immunological aspect on the developing fetus (e.g., hormones, cytokines), additional studies may tease out more direct outcomes of maternal stress in rodents and increase their translational value to human studies.

## Introduction

Maternal adversity during pregnancy influences neurodevelopment in human and model animal offspring (Goldstein et al., 2019; Sheng et al., 2021). Adversity can result from stressors coming from many different directions ranging from environmental to nutritional and physiological to immune (e.g., infection). Most stressors result in fetal overexposure to glucocorticoids [corticosterone in rodents, cortisol in humans] that have been directly linked to long- and short-term negative impacts on neurological health of offspring (Handa et al., 2022). Normally, the placenta protects the fetus from circulating glucocorticoids via the enzyme, 11β-Hydroxysteroid Dehydrogenase 2 (11β-HSD2). However, when there are sustained high levels of glucocorticoids, 11β-HSD2 cannot keep up the conversion of active to inactive glucocorticoids, leading to fetal overexposure (Welberg et al., 2000; Seckl and Holmes, 2007). Neuropsychiatric diseases postulated to have fetal origins are diverse and include such things cardiovascular disease, obesity, affective disorders, and metabolic and immune disorders (Goldstein et al., 2019).

Responses to stress are driven by components of the hypothalamic–pituitary–adrenal (HPA) axis. The neuroendocrine pathways and feedback loops of the HPA axis result in the stimulated release of glucocorticoids from adrenal glands (Dearing et al., 2022). As physiological modulators, glucocorticoids stimulate adaptative changes to physiological demands resulting from external stressors and to ultimately regain homeostasis. Regardless of the nature of maternal stress, one common critical impact is HPA axis activation and the release glucocorticoids that can influence developing fetuses. A frequently used model for evaluating fetal stress has been to bypass the complicated nature of the stressors and simply increase fetal glucocorticoid stimulation (Busada and Cidlowski, 2017). Given there is questionable access of endogenous glucocorticoids across placentas, studies often administer the synthetic glucocorticoid, dexamethasone (DEX) during periods of gestation. Evidence suggests that there may be negative long-term consequences of prenatal DEX exposure. Male and female offspring of rodent dams prenatally treated with DEX exhibit decreased body weights, sex-dependent changes in neuroendocrine and autonomic stress responses, and stress-related behavioral impairments (e.g., social-/anxiety-like behavior) (Zuloaga et al., 2012; Goldstein et al., 2014; Moisiadis and Matthews, 2014; Hiroi et al., 2016; Madhavpeddi et al., 2022; Rivet et al. 2022; Wang et al. 2022). However, prenatal DEX is a complicated model because DEX inhibits multiple aspects of the immune system and maternal immune activation is an important aspect of maternal stress. The current study investigated other approaches to promote inflammation that could influence the maternal-fetal environment (Smith and Reyes, 2017) and compare them to fetal DEX exposure.

Two frequent models of maternal stress include nutritional stress by high fat diet (HFD) or caloric restriction (CR) (Cortes-Albornoz et al., 2021; Van Gronigen Case et al., 2021). These exposures have been linked to behavioral alterations indicative of attention deficit hyperactivity disorder (ADHD), autism spectrum disorders (ASD), anxiety, and depression (Cortes-Albornoz et al., 2021; Wen et al., 2022; Castillo et al., 2023). In rodents, HFD during pregnancy increases anxiety-like behaviors in adolescent males and females. Inflammatory co-activators of the immune-stress axis (e.g., IL-6, NFkB, CD11b) were additionally increased in the hippocampus and amygdala with HFD in male and female juvenile offspring. These data indicate maternal nutritional stress alters neurobehavioral and immune responses in rodent offspring, but not all necessarily in a consistent manner between and even within the same models (Sasaki et al., 2014). Many prenatal caloric restriction (CR) studies in rodents show increased anxiety-like behavior in male and female adult offspring (Levay et al., 2008; Sheng et al., 2021), while others show decreased anxiogenic behavior in adult offspring (Govic et al., 2016; Fisch et al., 2019) or no changes in anxiety-like behavior with improved, memory performance, novelty-seeking and locomotor activity in adult male offspring (Lotufo et al., 2018).

Sex differences in the onset of neurodevelopmental disorders may arise due to the impact of gonadal steroid hormones, sex chromosomes or other gene-dependent mechanisms (Majdic and Tobet, 2011; Arnold, 2022). In rodents, many studies focus on behavior disorders after puberty, and there are less data available during the early adolescent periods when behavioral symptoms of adult neuropsychiatric disorders may begin to develop (Eltokhi et al., 2020). The current study examines prepubertal juvenile (28 days of age) male and female mice to determine whether sex differences develop prior to the emergence of significant pubertal sex steroids. The experiments in the current study directly compare the prenatal exposure to DEX model to nutritional stress models; maternal HFD and maternal CR. The effects of these modes of maternal stressors were examined to evaluate HPA stress responsiveness and stress-related behaviors in in prepubertal male and female mice. Social- and anxiety-like behaviors were assessed as our stress-related behavioral outputs since these disorders are thought to have fetal origins influenced by maternal stress (Sheng et al., 2021).

## Methods

### Maternal stress models

**Figure 1:**
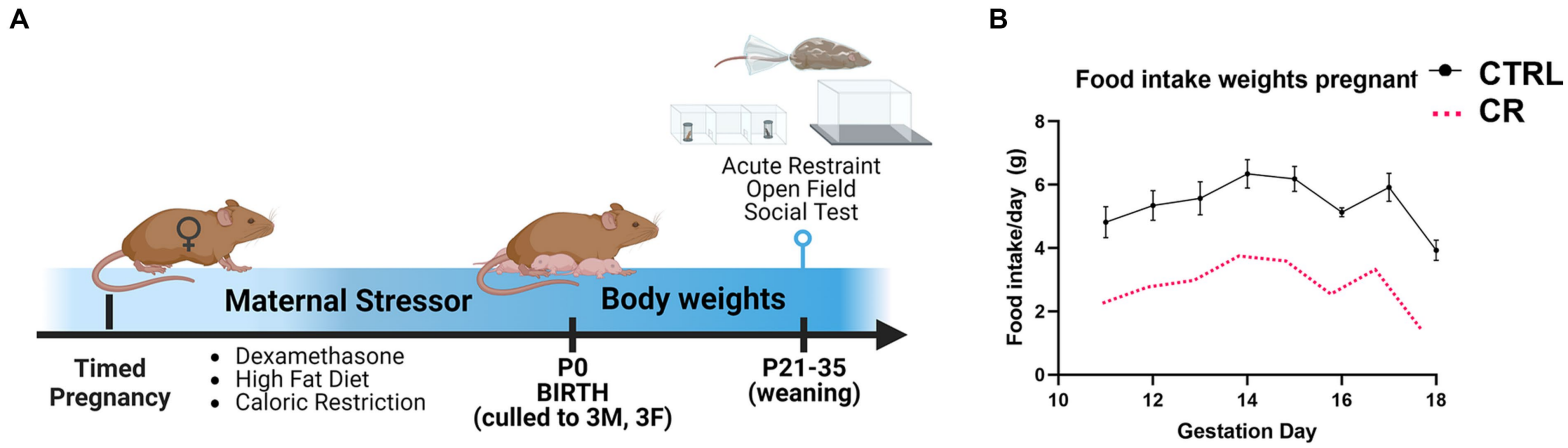
Experimental timeline. **(A)** Pregnant females were randomly assigned to one of the following types of maternal stress model: injection of dexamethasone (DEX; gestation day 15–18), high fat diet (HFD; gestation day 0 to parturition), or caloric restricted diet (CR). **(B)** The CR diet was 50% of the average food intake of the control (CTRL) females from gestation day 11 to parturition. *N* = 6–8dams/treatment, 1–2 mice/sex used for each outcome.

Timed-pregnant C67B/6 N females were exposed to one of the following types of maternal stress described below (*N* = 8 dams/treatment and control group for each maternal stressor; each model of maternal stress had its own control group). All litters were culled to 3 females and 3 males at birth. These methods maintained controlled litter size of 6 and prevent variability in nutritional differences from biasing data. One to two offspring of each sex per dam was used in each treatment group, with a total of 7–9 mice/sex in each group. Offspring were weaned at postnatal day 21 to begin testing for anxiety-like and social-like behaviors, followed by acute physical restraint to examine corticosterone responses to stress (Figure 1). Pups were weighed weekly until euthanasia. Mice were housed with *ad libitum* access to food (unless otherwise stated) and water and on a 12:12 light: dark cycle (lights on at 06:00 and off at 18:00). Restraint, blood collections, and behavior testing were performed during 09:00 and 15:00 to avoid diurnal elevations in plasma corticosterone. Behavior assays were performed from least stressful to most, ending with restraint. All subject animals were gonadally intact at the time of testing. All mice were euthanized by inhalation of 30–70% carbon dioxide delivered in a sealed chamber until breathing ceased, consistent with Colorado State University’s Institutional Animal Care and Use Committee and AVMA Euthanasia Guidelines. This was followed by exsanguination by intracardial perfusion with phosphate saline buffer and 4% buffered paraformaldehyde to fix tissues or decapitation using sharp scissors according to AVMA approved methods.

## Ethics statement

All procedures were approved by Colorado University Lab Animal Resources and Institutional Animal Care and Use Committee Guidelines (Protocol CoA #: 1657).

### Dexamethasone

Timed-pregnant females treated with dexamethasone dissolved in 25% beta-cyclodextrin in 0.9% saline (Subcutaneous injection; 0.4 mg/kg) or vehicle (25% beta-cyclodextrin in 0.9% saline, control group) on gestation days 15–18. The route and dosage of injection was based on our prior studies dosage was optimized for consistency with clinical administration of DEX to pregnant women (Zuloaga et al., 2012; Hiroi et al., 2016; Madhavpeddi et al., 2022).

### High fat diet

Timed-pregnant females placed on control diet (Envigo TD.2919) or 60% high fat diet (Envigo TD.06414) from beginning of pregnancy (gestation day 0) to parturition (Christians et al., 2019). Females were switched back to control diet after birth to selectively tease out behavioral outputs due to nutritional stress *during* gestation without the confounding variable of the rearing/lactation period.

### Caloric restriction

Timed-pregnant females underwent caloric restriction compared to controls from gestation day 11 to parturition (Christians et al., 2021). Daily food intake was measured in control pregnant dams (in grams) and then caloric restricted dams received 50% of the measured amount (Figure 1). Control dams were allowed *ad libitum* access to food. Both groups were allowed *ad libitum* access to water. All dams were placed back on *ad libitum* access to food and water at parturition to parse out effects due to effects *during* gestation without potentially altering behavioral outputs that can occur if the mother was kept caloric restricted throughout lactation. Cannibalism of neonate pups was also tracked, with no significant differences between caloric restricted and control mothers.

### Behavioral assays

#### Social interaction test

Subjects were placed in a 3-chamber apparatus with an empty wire-mesh cage on opposing ends as previously described (Borrow et al., 2019). Subject were then allowed to habituate in the apparatus. After 10 min, an unfamiliar age- and sex-matched stimulus mouse was placed under a wire-mesh cage. The opposing wire-mesh cage remained empty. To examine social discrimination behavior of juvenile offspring, we measured the time spent investigating stimulus mice versus empty wire-mesh cage. The cups and 3-chamber apparatus were cleaned with 70% ethanol and dried prior to and following each test. All behavior trials were video-recorded, and analysis and animal position tracked by Ethovision software (Noldus Information Technologies). Sociability was analyzed by examining the duration and frequency of visits the subject mice spent investigating the wire-mesh cage containing the novel stimulus mice. Social interaction test results were analyzed statistically using a two-way ANOVA (maternal stress X offspring sex) using GraphPad Prism (GraphPad Software, La Jolla, CA).

#### Open field

Activity was assessed by placing an experimental mouse in the center of a circular open field area made of Plexiglass (height = 30 cm, radius = 20 cm) as in previous studies (Oyola et al., 2012). Mice were left undisturbed for 10 min and returned to their home cage following the test. Tests were performed on one mouse at a time. The arena was washed with 70% ethanol and water and dried to eliminate odors between each subject. The time spent in the center ring and total distance traveled were measured. The total time spent in the outer ring (closer to the wall of the arena) was also measured, demonstrating the same output in anxiety-like behavior trends as the time spent in the center ring (data not shown).

### Corticosterone measurements and acute restraint

Offspring underwent 20 min acute restraint inside a spatially constricted tube with 60 min recovery in their home cage. Restraint is considered a mild psychological stress. The animal was not completely immobilized, but movement was restricted in restraint tube. A breathing hole was located at the end of the tube where the nose reaches. The animal had no more than a centimeter of movement on all sides. There were also holes along the lateral sides of the restraint tube for more adequate ventilation. All restraint stress was performed between 09:00 and 14:00 to avoid diurnal elevations in corticosterone (Miller et al., 2022). Tail blood was collected at three timepoints during restraint: 0 min (prior to restraint), 20 min immediately after acute restraint, and 60 min after released from restraint and allowed to recover in home cage. About 15 microliters of blood will be collected to obtain at least 10 microliters of plasma for enzyme-linked immunosorbent assay. After the 60 min recovery in home cage, the animal was anesthetized by inhalation of 30–70% carbon dioxide delivered in a sealed chamber until breathing is ceased and euthanized by intracardial perfusion (Frahm and Tobet, 2015). Plasma corticosterone levels were measured by Enzyme-Linked ImmunoSorbent Assay (ELISA) per manufacturer’s guidelines (Arbor Assays, Ann Arbor, MI; cat no. K014-H1; Limit of detection 7.7 pg./mL mean intra-assay CV = 8.5%). Briefly, tail vein blood (collected at the 3 timepoints described above) was placed into chilled tubes with 0.5 M EDTA and aprotinin (4 mg/mL, Sigma-Aldrich, St. Louis, MO), then centrifuged in a Beckman J6 centrifuge at 2,000 rpm at 4°C for 10 min. After the plasma was separated, it was stored at −20°C until assayed. At the beginning of the assay, plasma samples (5 μL plasma per sample well, every sample was run in duplicate) were prepared with Dissociation Reagent to dissociate the corticosterone from corticosteroid binding globulin. A standard curve was prepared from increasing dilutions (5,000, 2,500, 1,250, 625, 312.5, 156.25, 78.125, 39.063, and 19.531 pg/mL) of corticosterone. DetectX Corticosterone Conjugate and DetectX Corticosterone Antibody was added to each well. Tetramethylbenzidine solution was next added to each well. The optical density of each sample was determined with a wavelength of 450 nm in Azure biosystems Ao microplate reader (Azure Biosystems, Inc., Dublin, CA). To calculate the concentration of corticosterone, the duplicate optical density readings for each standard and sample were averaged. A standard curve was generated using the online tool from “MyAssays” through Arbor Assays. The sample concentrations were calculated from the %B/B0 curve and multiplied by the dilution factor to obtain the neat sample values.

### Statistical analysis

Results are presented as means ± sem. Data were analyzed using Prism (GraphPad Software Inc., La Jolla, CA). For each significant three-way ANOVA, *post hoc* comparisons were made using Dunn’s method for the comparison of all groups vs. the control group or Fisher Least Significant Difference test for multiple comparisons. Results for corticosterone assay were analyzed statistically by three-way ANOVA (Treatment X restraint x sex) using GraphPad Prism (GraphPad Software, La Jolla, CA). Bonferroni’s correction factor was used for post-hoc analysis. Significance was set at *p* < 0.05.

## Results

### Maternal treatment with dexamethasone

**Figure 2:**
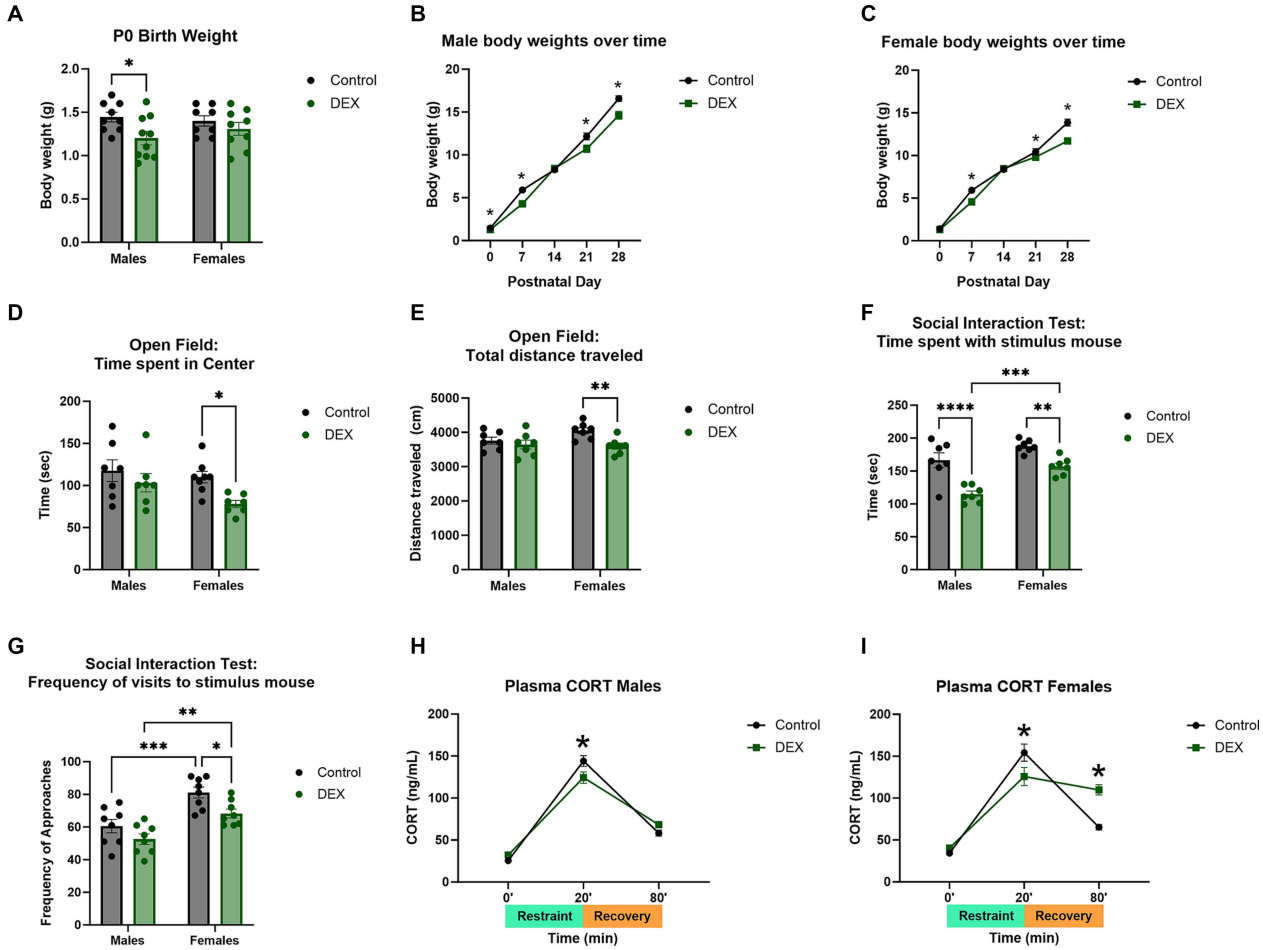
Maternal dexamethasone (DEX) resulted in altered body weights, behavior, and stress response in prepubertal male and female offspring. **(A)** Neonatal body weights at postnatal day 0 (P0) were decreased in males, but not females, exposed to prenatal DEX. Over time **(B)** males and **(C)** females demonstrated decreased body weights from P7–P28. Open field assay revealed increased anxiety-like behavior in females as measured by **(D)** time spent in the center and **(E)** total distance traveled. No changes were seen in males. Social-like behavior was impaired in offspring. Male and female pups exhibited decreased **(F)** time spent with and **(G)** and frequency of visits to stimulus mouse. Acute stress response was decreased in **(H)** males and **(I)** females exposed to prenatal DEX immediately after 20 min of restraint as measured by plasma corticosterone. Prenatal DEX females further exhibited prolonged increases of corticosterone at the 80 min timepoint (60 min after removal from restraint), indicating delayed negative feedback to HPA axis stress response. *p < 0.05, **p < 0.01, ***p < 0.001, and ****p < 0.0001 DEX vs. Control.

There were several characteristic changes in juvenile male and female offspring from mothers treated with DEX during the last 4 days of gestation. Weekly assessment of body weights in offspring from treated mothers indicated a decrease related to maternal DEX that was noted selectively in males (Figures 2A–C) [*F* (1, 32) = 6.002, *p* = 0.02]. Using an open field assay to assess anxiety-like behavior, 2-way ANOVA revealed female, but not male offspring, from mothers exposed to prenatal DEX, exhibited less time spent in the center ring and total distance traveled compared to controls (Figures 2D–E; *p* < 0.01). This implies increased anxiety-like behavior selectively in female offspring of mothers exposed to DEX during gestation. Social behavior was impaired in offspring of mothers treated with DEX [*F* (1, 28) = 9.591, *p* = 0.004] in both sexes (Figures 2F–G). Post-hoc analysis revealed male (*p* < 0.01) and female (*p* < 0.01) offspring of mothers treated with DEX spent less time investigating stimulus mice. Prenatal DEX offspring also exhibited a lower frequency of visits to stimulus mice compared to control offspring (*p* < 0.01). Plasma corticosterone levels as a measure of HPA axis stress responsiveness altered by prenatal DEX treatment in males [*F* (2, 36) = 269.1, *p* < 0.0001] and females [*F* (2, 39) = 116.1, *p* < 0.0001]. Peak levels were lower after 20 min of acute restraint stress in prenatal DEX exposed males (*p* < 0.05) and females (*p* < 0.05) compared to control offspring (Figures 2H–I). Interestingly, plasma corticosterone was still elevated 60 min after restraint in female offspring from DEX treated mothers versus controls (*p* < 0.001).

### Maternal dietary manipulations (high fat diet and caloric restriction)

**Figure 3:**
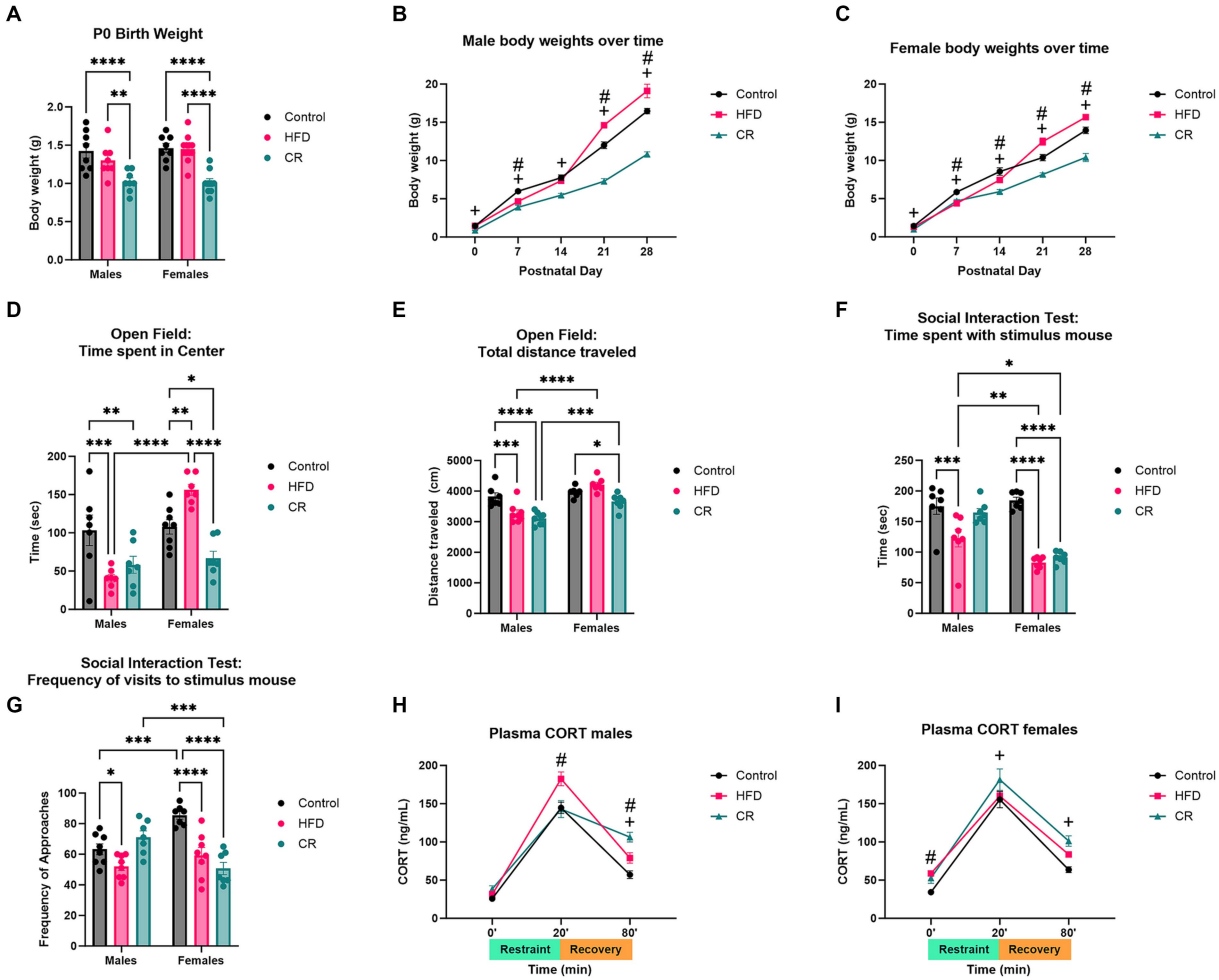
Maternal dietary manipulation (high fat diet and caloric restriction) resulted in altered body weights, behavior, and stress response in prepubertal male and female offspring. **(A)** Neonatal body weights were unchanged in high fat diet (HFD) but decreased in CR offspring pups. Over time, **(B)** male and **(C)** female offspring exposed to maternal HFD increased in body weight compared to controls, while caloric restriction (CR) offspring decreased in weight. Anxiety-like behavior was altered by diet manipulation. HFD and CR males showed increased anxiety-like behavior in open field assay, as measured by **(D)** time spent in center and **(E)** total distance traveled. Only CR females exhibited increased anxiety-like behavior with decreased **(D)** time spent in center and **(E)** total distance traveled in open field test. Social behavior was impaired in HFD males and females and CR females with decreased **(F)** time spent with and **(G)** frequency of visits to stimulus mouse. Plasma corticosterone levels were increased in **(H)** HFD males after 20 min of restraint. HFD and CR males both demonstrated prolonged elevations of corticosterone after 60 min recovery. **(I)** HFD females showed increased baseline corticosterone levels while CR females had elevated levels after 20 min of restraint and 60 min recovery. *p < 0.05, **p < 0.01, ***p < 0.001, and ****p < 0.0001 vs. Control. ^#^HFD vs. Control. ^+^CR vs. Control.

Offspring from mothers on HFD did not show significant differences in neonatal body weights compared to controls. Body weights of CR male [*F* (1, 104) = 163.6, *p* < 0.0001] and female [*F* (2, 93) = 79.21, *p* < 0.0001] offspring (Figures 3A–C) were significantly lower than both control and HFD groups. After weaning, juvenile pups exhibited alterations in anxiety-like and social-like behaviors (Figures 3D–G). In open field assays, offspring maternal dietary manipulation increased anxiety-like behavior measured by time spent in center ring [*F* (2, 37) = 8.391, *p* < 0.0001] and total distance traveled [*F* (2, 38) = 15.27, *p* < 0.0001]. Post-hoc analysis revealed HFD and CR males showed less time spent in center ring (*p* < 0.01) and less total distance traveled (*p* < 0.01) compared to controls (Figures 3D–E). In contrast, female offspring from mothers fed HFD showed lower anxiety-like behavior with greater duration of time spent in center ring (*p* < 0.0001) while female offspring from CR mothers demonstrated less time spent in center (*p* < 0.0001 vs. Control) and distance traveled (*p* < 0.05 vs. Control). Such data suggest more anxiety-like behavior with maternal exposure to CR. Social interaction testing additionally revealed effects of diet [*F* (2, 39) = 13.09, *p* < 0.0001]. Male (*p* < 0.01) and female (*p* < 0.0001) offspring from mothers fed HFD exhibited impaired social behavior with less time investigating the stimulus mouse compared to controls (Figures 3F–G). Interestingly, only female offspring from mothers fed HFD showed fewer visits to stimulus mice (Figures 3F–G). Male offspring from mothers fed HFD also spent increased time investigating the empty cage (*p* < 0.001) with more visits to the empty cage (*p* < 0.005) while females did not when compared to their same-sex controls (Figures 3F–G). Comparatively, female offspring from CR mothers, but not male offspring showed impaired social behavior with less investigatory behavior (*p* < 0.0001) and number of visits (*p* < 0.0001) to stimulus mice (Figures 3F–G). Maternal HFD and CR further elevated acute stress response in female [(*F* (2, 56) = 11.56, *p* < 0.0001)] and male [*F* (2, 52) = 9.175, *p* < 0.0004] offspring. Stress-induced plasma corticosterone levels were elevated at baseline in female offspring of HFD mothers (*p* < 0.05) compared to controls. After 20 min acute restraint stress, plasma corticosterone was higher only in male offspring from HFD mothers (*p* < 0.001) compared to controls. Similarly, corticosterone continued to be elevated in a male-biased manner after 60 min recovery in offspring from HFD mothers compared to controls (Figures 3H–I). In female offspring of CR mothers, plasma corticosterone was higher after 20 min restraint compared to controls (*p* < 0.05), and was still elevated in CR and HFD male (*p* < 0.0001 vs. controls) and female (*p* < 0.001 vs. controls) offspring after 60 min.

## Discussion

The current study examined effects of three types of maternal stressors on male and female offspring assessed in pre-pubertal mice. Behaviors were examined prior to puberty because of the potential hormone dependence of the onset of neuropsychiatric disorders. Behaviors or biomarkers that might show sex differences prior to the emergence of significant hormone secretion at puberty could be helpful for predicting the onset of disorder since many neuropsychiatric disorders are diagnosed during early juvenile years in humans (Kim-Cohen et al., 2003). The results demonstrate birth weight, weight gained over time, stress response, and stress-related behaviors (anxiety- and social-like behaviors) were altered differentially with some similarities depending on the model of maternal stress exposure given the different timings (early, middle, late gestation), type (glucocorticoid, metabolic), and duration (part- or entirety- of gestation) of exposure (Table 1).

**Table 1:** Brief description of changes in male (♂) and female (♀) offspring exposed to dexamethasone (DEX), caloric restriction (CR), or high fat diet (HFD) stress during pregnancy.

Maternal Treatment	DEX		CR		HFD	
	♂	♀	♂	♀	♂	♀
P0 body weight	↓	–	↓	↓	–	–
Anxiety-like (OF)	–	↑	↑	↑	↑	↓
Social	↓	↓	–	↓	↓	↓
Basal CORT at 0’	–	–	–	↑	–	↑
CORT after 20’ acute stress	↓	↓	–	–	↑	↑
CORT after 60’ recovery	–	↑	↑	↑	↑	–

Increase (↑), Decrease (↓), and No change (–).

### Prenatal dexamethasone exposure

Fetal exposure to the synthetic glucocorticoid, DEX, altered development of the offspring. In agreement with other studies in rodents, body weights were lower in offspring treated with DEX during late gestation (Carbone et al., 2012). In the current study there was more anxiety-like behavior using an open field behavior assay with less time spent in center ring and total distance traveled selectively in female offspring. This agrees with other studies that demonstrated adult female offspring to be more susceptible than males after prenatal exposure to glucocorticoids (Hiroi et al., 2016). Specifically, timing of fetal exposure to elevated glucocorticoids has been implicated in sex differences in the developing offspring. In rats, earlier exposure has demonstrated male-specific effects on the HPA axis stress response, while exposure later in gestation has shown greater effects in females (Kamphuis et al., 2002; Carbone et al., 2023). Levels of baseline corticosterone and ACTH were found to be elevated, accompanied by greater anxiety- and depressive-like behaviors, in selectively females exposed to late gestation glucocorticoids (Richardson et al., 2006; Hiroi et al., 2016). Interestingly, male and female offspring from mothers injected with DEX exhibited social impairments in the social interaction test. Many reports suggest prenatal glucocorticoid exposure by treatment significantly influences brain regions involved in the HPA axis stress response, including amygdala and hippocampus (de Kloet et al., 2005; Colciago et al., 2015). These regions are also involved in stress-related outputs, including cognitive, sociability, and memory function. Several studies demonstrate high expression of mineralocorticoid and glucocorticoid receptors, making regions, such as the amygdala and hippocampus, susceptible to excess exogenous glucocorticoids, such as DEX (Colciago et al., 2015). Prenatal exposure to DEX could influence the development of these areas in the brain, perhaps by altering receptor function or morphology. More studies are needed to better examine these mechanisms. In the current study, female mice, but not males, exhibit dysregulation of HPA axis reactivity with sustained elevation in plasma corticosterone following acute stress. Another study that administered prenatal DEX to pregnant mice dams during mid-late gestation (gestation days 11–17) reported greater corticosterone stress response in female offspring from DEX-treated mothers (Frahm et al., 2018). Even though the stressor began earlier in gestation, DEX was administered throughout the end of late gestation, which seems to be a critical timepoint of fetal development for sex-dependent effects. These sex differences in stress responses and related behaviors could potentially be used as early indicators to better predict the emergence of affective disorders after puberty. There is, however, a significant limitation of the use of DEX as a maternal stressor in that it is an incomplete model of stress. Injection of a synthetic glucocorticoid mimics one aspect of a stress response but it can potentially down-regulate other aspects of a normal stress response. Therefore, we explored additional models of maternal stress, i.e., under- and over-nutrition during pregnancy.

### Prenatal nutritional stress (HFD, CR) exposure

In the current study, nutritional stress during pregnancy altered body weights, anxiety- and social-like behavior, and HPA axis reactivity (Table 1). Animal models of maternal nutritional stress impacted fetal neural programming, leading to increased risk for developmental disorders, including early onset of ASD- and ADHD-like symptoms and those that emerge later, such as anxiety- and depressive-like symptoms (Cortes-Albornoz et al., 2021; Castillo et al., 2023). Offspring from clinically obese mothers have increased risk for severe ADHD and ASD symptoms pre-pubertally (Neri and Edlow, 2015; Castillo et al., 2023; Sybock et al., 2023) while those exposed to nutrient deficiencies are two times more susceptible in developing schizophrenia later in adulthood (Brown and Susser, 2008; Castillo et al., 2023). Rodent models of maternal obesity commonly use a HFD during pregnancy to mimic diets of Western societies. Studies have shown maternal HFD produces offspring that exhibit increased anxiety-like behavior in adult males and females. In the current study, juvenile males selectively showed greater levels of anxiety-like behavior in open field, suggesting male offspring are more susceptible to behavioral changes caused by a maternal HFD than females. This male-dependent effect is consistent with previous findings indicating maternal HFD programs the HPA axis and increases anxiety-like behavior in male offspring (Grundwald and Brunton, 2015; Lin et al., 2023). One study also demonstrated a maternal HFD impairs hippocampal function, an upstream regulator of HPA axis function, and increased anxiolytic-like behavior in in male offspring only (Bale, 2011; Brunton, 2015). Such findings suggest males at higher risk for anxiety-like disorders with exposure to HFD during pregnancy when the dietary manipulation was throughout the pregnancy (and differs from female selective DEX treatment effect that was limited to gestation day 15–18 or maternal CR from gestation day 11-parturition). Future studies need to be done to determine whether this effect is influenced more by male gonadal sex hormones (androgens) or sex chromosomes (Arnold, 2014). In mice, testes develop around embryonic day 12.5 (Payne and Youngblood, 1995) and testosterone levels rise more in males than females by embryonic day 16 (Pointis et al., 1979; vom Saal and Bronson, 1980). Sex chromosome affects could occur at any point. Social-like behavior was also impaired in male and female offspring (Sullivan et al., 2012, 2015). In agreement with the current results, other studies have shown impaired social interaction in mouse offspring exposed to maternal HFD (Kang et al., 2014; Sgritta et al., 2019). One study correlated such social deficits with increased proinflammatory cytokines in the brain and a female-specific increase in microglial activation (Kang et al., 2014). There is growing evidence that proinflammatory cytokines, such as interleukin-1β and Tumor Necrosis Factor α, are associated with altered cognitive and social function (Bilbo et al., 2005; Terrando et al., 2010). Higher levels of cytokines in the brain could be a result of greater microglia activity, but more experiments are necessary to test this hypothesis.

Fetal exposure to maternal HFD elevated HPA axis stress reactivity, altering social-like and aggressive-like behaviors in offspring (Sullivan et al., 2012, 2015). The current study examined HPA axis function in response to an acute restraint stressor. Data show the HPA axis stress response was greater in offspring exposed to maternal HFD with a delayed return to baseline glucocorticoid levels. This could be due to increased expression of glucocorticoid receptors in other brain regions that project to the hypothalamus to stimulate the stress response, such as hippocampus and amygdala (Sasaki et al., 2014). Other studies demonstrate maternal HFD increases basal corticotropin releasing hormone expression in the hypothalamus, the main neuropeptide that responds to stressful stimuli to trigger the HPA axis cascade and increase glucocorticoid secretion (Niu et al., 2019). Studies are needed to pinpoint specific mechanisms by which a maternal HFD programs HPA axis in offspring and how these programming effects lead to social- and anxiety-like impairments.

Under-nutrition models can range from mild restriction of food intake (10–15%) to more moderate restricted food intake (50–75%) (Grissom et al., 2017). The current study consisted of more moderate restricted food intake (50%) over the last week of pregnancy, but still resulted in increased social/anxiety-like symptoms and a hyperactive stress response in offspring. Interestingly, maternal CR resulted in social deficits selectively in female offspring. This could be largely associated with higher basal corticosterone levels in females, suggesting an overactive HPA axis at baseline. In another experiment, maternal CR led to hyperactivation of the HPA axis in both dams and offspring (Martins et al., 2023). Hyperactivation of the stress axis in dams could robustly influence maternal behavior, where poor maternal care can negatively impact neural development in offspring and lead to increased anxiety- and social-like behaviors. While the current results are in agreement with several maternal feeding restriction studies (Levay et al., 2008; Akitake et al., 2015; Govic et al., 2016; Van Gronigen Case et al., 2021), other studies show maternal CR (undernutrition during pregnancy) results in decreased in anxiety/social-like behavior in prepubertal offspring (Grissom et al., 2017). These differing results could be explained by varying restricted food intake regimens, such as restriction during all of gestation, from parturition throughout lactation, or during both gestation and lactation (Levay et al., 2008; Akitake et al., 2015; Fisch et al., 2019). Another reason for varying results could be that many studies investigate behavioral changes in older adult offspring exposed to caloric restriction during fetal life. Juvenile, pre-pubertal mice can exhibit different behavior than adult post-pubertal mice, such as altered social- or anxiety-like and locomotor behavior in social interaction test and open field assays (Eltokhi et al., 2020, 2021). Many mice studies that examine neuropsychiatric-like symptoms are in adults. However, the current study demonstrates the feasibility of mimicking diagnoses of neuropsychiatric-like symptoms in juvenile mice, emphasizing the importance of behavioral testing early on in preclinical models.

### Summary

Extensive evidence shows that perturbations to fetal environments cause long-term consequences to HPA axis physiology, increasing risk for related disorders in adulthood. The idea that glucocorticoid responses to different stressors might serve as a common stimulus across stress paradigms is insufficient, given that different modes of prenatal stress may produce differential effects as we showed in our study. Opposite nutritional stressors produced similar outcomes for anxiety-like behavior in both sexes, social-like behavior in females, and a hyperactive adrenal stress response in males. The timing and duration of exposure to maternal stressor also heavily influences the outputs because several organs and tissues develop at different critical periods during gestation (Bayer et al., 1993). Such programming effects can lead to sex-dependent behavioral and neuroendocrine outcomes due to the timing of development and maturation of sex gonads (and subsequent gonadal steroid production) and sexual dimorphic regions of the brain (Brunton et al., 2015). Studies suggest maternal insults during certain critical periods during fetal development influences sex-specific effects on HPA axis development, influencing stress response and related behaviors. One critical period of HPA axis development occurs during late gestation in rodents, when male rodents are exposed to a surge of testosterone that can contribute to brain sexual differentiation (Ward, 1984). Fetal exposure to maternal stressors during this time could alter the testosterone surge, leading to improper HPA axis development and function in postnatal life (Ward and Weisz, 1984). More molecular studies in the context of key brain regions (e.g., paraventricular nucleus in hypothalamus or nucleus of the solitary tract in the brainstem) are needed to tease apart specific mechanisms involved in the testosterone surge in the male fetus and maternal stress.

Of note in the current study, the 3 treatments were overlapping in their timing, and different in their duration. HFD was from the beginning of the pregnancy, CR from embryonic day 11, and DEX from embryonic day 15. Additionally, perturbations in the postnatal environment also play a role in susceptibility for neuropsychiatric disorders. This addresses the idea of a “dual hit” hypothesis, where the compounding effects of both a pre- and postnatal stressor (e.g., during lactation prior to weaning) increases risk for disease later in life even more (Nabeshima and Kim, 2013; Verstraeten et al., 2019; Guerrin et al., 2021). In the current study, the offspring were not cross fostered at birth and potential maternal behavior changes were not evaluated.

One common theme among the three models presented here (DEX, CR, HFD) is that there are consistent reports demonstrating their role in activating the maternal and fetal immune responses (Hale et al., 2015; Goldstein et al., 2016; Edlow, 2017; Frahm et al., 2018; Edlow et al., 2019). Prenatal adversity in humans is shown to lead to elevated glucocorticoids and cytokines in the maternal environment (Goldstein et al., 2016). These alterations in the maternal environment can lead to changes in the fetus (impaired brain function, neuronal cell death, irregular hormone and cytokine levels, etc.,) that may serve as fetal antecedents for neurological disorders in adulthood (Goldstein et al., 2016). By focusing more granularly on the immediate physiological impact of different stressors on developing fetuses (e.g., hormones, cytokines), future studies may be able to parse out the different longer-term outcomes of prenatal stress models in rodents and their relationship to outcomes in humans.

## Data availability statement

The raw data supporting the conclusions of this article will be made available by the authors, without undue reservation.

## Ethics statement

The animal study was approved by Lab Animal Resources at Colorado State University, Institutional Animal Care and Use Committee. The study was conducted in accordance with the local legislation and institutional requirements.

## Author contributions

JS: Conceptualization, Data curation, Formal analysis, Investigation, Methodology, Project administration, Writing – original draft, Writing – review & editing. RH: Conceptualization, Funding acquisition, Resources, Writing – review & editing. ST: Conceptualization, Funding acquisition, Resources, Supervision, Writing – review & editing.
